# On the Track of the Missing tRNA Genes: A Source of Non-Canonical Functions?

**DOI:** 10.3389/fmolb.2021.643701

**Published:** 2021-03-16

**Authors:** Ricardo Ehrlich, Marcos Davyt, Ignacio López, Cora Chalar, Mónica Marín

**Affiliations:** ^1^ Biochemistry-Molecular Biology, Faculty of Science, Universidad de la República, Montevideo, Uruguay; ^2^ Institut Pasteur de Montevideo, Montevideo, Uruguay

**Keywords:** missing tRNAs, tRNA functions, tRNA modifications, tRNA interactions, non-canonical tRNA functions, tRNA structure

## Abstract

Cellular tRNAs appear today as a diverse population of informative macromolecules with conserved general elements ensuring essential common functions and different and distinctive features securing specific interactions and activities. Their differential expression and the variety of post-transcriptional modifications they are subject to, lead to the existence of complex repertoires of tRNA populations adjusted to defined cellular states. Despite the tRNA-coding genes redundancy in prokaryote and eukaryote genomes, it is surprising to note the absence of genes coding specific translational-active isoacceptors throughout the phylogeny. Through the analysis of different releases of tRNA databases, this review aims to provide a general summary about those “missing tRNA genes.” This absence refers to both tRNAs that are not encoded in the genome, as well as others that show critical sequence variations that would prevent their activity as canonical translation adaptor molecules. Notably, while a group of genes are universally missing, others are absent in particular kingdoms. Functional information available allows to hypothesize that the exclusion of isodecoding molecules would be linked to: 1) reduce ambiguities of signals that define the specificity of the interactions in which the tRNAs are involved; 2) ensure the adaptation of the translational apparatus to the cellular state; 3) divert particular tRNA variants from ribosomal protein synthesis to other cellular functions. This leads to consider the “missing tRNA genes” as a source of putative non-canonical tRNA functions and to broaden the concept of adapter molecules in ribosomal-dependent protein synthesis.

## Introduction

“Though this be madness, yet there is method in it.”

(Hamlet Act 2, scene 2)

Over the last few years, tRNA has become the subject of intense research where technological and conceptual advances converge from integrative biology to pathology and biotechnology. An extraordinary volume of work has led to characterize the population of tRNAs in organisms that cover the entire phylogeny, both by analysis of tRNA molecules and genomic data (Chan and Lowe, 2009; Jühling et al., 2009; Lowe and Chan, 2016). Yet, it is interesting to notice that tRNAs, which have been the first sequenced nucleic acid molecule (Holley et al., 1965) and the first solved three-dimensional structure (Kim et al., 1974), still pose complex challenges centered on their structure and functions, despite the remarkable advances in sequencing techniques and structural studies of nucleic acids (Dittmar et al., 2006; Pang et al., 2014; Ferro and Ignatova, 2015; Zhang et al., 2015; Evans et al., 2017; Shigematsu et al., 2017; Kimura et al., 2020; Pinkard et al., 2020; Zhang et al., 2020). Old questions that focus on the structure-function relationship and fidelity of the interactions and cellular processes in which these molecules participate are thus renewed. Simultaneously, the enormous amount of information that has flourished in the last decade updates evolutionary questions, whose projections acquire new implications in the understanding of numerous biological processes and are related to a vast field of applications (Torres et al., 2014a; Kirchner and Ignatova, 2015; Orioli, 2017; Ho et al., 2018; Tharp et al., 2020).

As central adapter molecules in protein biosynthesis, the diversity of tRNA isoacceptors or isotypes (tRNA genes or tRNA molecules with different anticodon that charge the same amino acid), isodecoders (molecules with the same anticodon but different body structure), and modified states thereof, ensures the transmission of information from a nucleotide to an amino acid sequence. tRNAs also participate in the adjustment of the translation machinery and its kinetics, as well as in co-translational folding of peptides in order to meet the cellular requirements in terms of repertoire and relative amount of proteins (Gingold et al., 2014; Kirchner and Ignatova, 2015; Kirchner et al., 2017; Marín et al., 2017; Rak et al., 2018). Moreover, they play an important role in a number of adaptive processes associated with changes in cellular programs, including major metabolic options, non-ribosomal protein synthesis, and a large number of regulatory mechanisms, that are part of the so-called non-canonical functions.

Cellular tRNAs appear today as a diverse population of informative macromolecules with conserved general elements ensuring essential common functions and different and distinctive features securing specific interactions and activities. Their differential expression and the variety of post-transcriptional modifications they are subject to, lead to the existence of complex repertoires of tRNA populations adjusted to defined cellular states (Dittmar et al., 2006; Pavon-Eternod et al., 2009; Gingold et al., 2014; Pang et al., 2014; Goodarzi et al., 2016; Sagi et al., 2016; Kimura et al., 2020; Pinkard et al., 2020). Moreover, an important redundancy has been described in tRNA-coding genes (tDNAs) both in prokaryotes (in some cases up to a hundred) and in eukaryotes (several hundreds) (Goodenbour and Pan, 2006; Fujishima and Kanai, 2014; Chan and Lowe, 2016; Pan, 2018; Rak et al., 2018). As algorithms that identify tRNA genes were improved, the number of tDNAs annotated using high stringency criteria decreased (GtRNAdb http://gtrnadb.ucsc.edu/ data release 18:1, August 2019). This allowed to detect the complete or almost complete absence of genes coding for specific isoacceptors, in either all kingdoms or specifically in bacteria, archaea or eukaryotes. In that sense, expressions drawing the attention to this observation, such as “pseudo-tRNAs,” “silent genes,” “absent genes,” “deleterious,” “toxic,” and “prohibited tRNA species,” appeared in the scientific literature (Maraia and Arimbasseri, 2017; Rak et al., 2018; Torres, 2019; Pernod et al., 2020). On the other hand, evidence has also accumulated on the existence of particular species of isoacceptor tRNAs for specific amino acids, defined according to their structural characteristics and anticodon, with different functionalities (Giannouli et al., 2009; Rudinger-Thirion et al., 2011; Rogers et al., 2012; Torres, 2019).

This review is intended to shed light on a particular point: the astonishing absence of genes encoding functional isoacceptor tRNAs carrying particular anticodons throughout the phylogeny. This absence not only refers to tRNAs that are not encoded in the genome but also to others that have critical sequence variations that impair their activity as canonical translation adaptor molecules. Indeed, the analysis of genomic (Lowe and Chan, 2016; Chan and Lowe, 2019) and directly sequenced tRNA databases (Jühling et al., 2009), reveal that prokaryotes and eukaryotes lack an important number of putative tRNAs bearing particular anticodons. This observation has been pointed out by several authors, and has been associated with different aspects including genetic code origin, evolution, and disambiguation mechanisms ensuring the fidelity of the different translation steps (Maraia and Arimbasseri, 2017; Diwan and Agashe, 2018; Rak et al., 2018; Lei and Burton, 2020). Although a systematic and integrated overall perspective about the absence of tDNAs is progressively emerging, much work is still required. This review aims to summarize a general view about the “missing tRNA genes” and to emphasize open questions about tRNA structure-function relationship and their multiple cellular functions.

This work is based on the analysis of different releases of tRNA databases. It should be mentioned that while the absence of a tDNA constitutes a fair evidence, the identification of a putative tRNA coding sequence is not a definite proof of the presence of a tRNA able to be aminoacylated and functionally active in translation. Thus, manual curing was carried out when required, following the criteria summarized by Marck and Grosjean (2002) and Giegé et al. (2012) to consider a sequence to be able or not to fold into a canonical “L-shape” structure. It should also be kept in mind that -with exceptions- there is scarce information available across the phylogeny about the transcription of different genes and the functionality of the encoded molecules. This is because albeit significant recent improvements (Zhang et al., 2015; Kimura et al., 2020), there are still technical restrictions to identify transcription products of specific putative tDNAs due to the presence of numerous post-transcriptional modifications that interfere with sequencing-by-synthesis techniques and hybridization methodologies.

**FIGURE 1:**
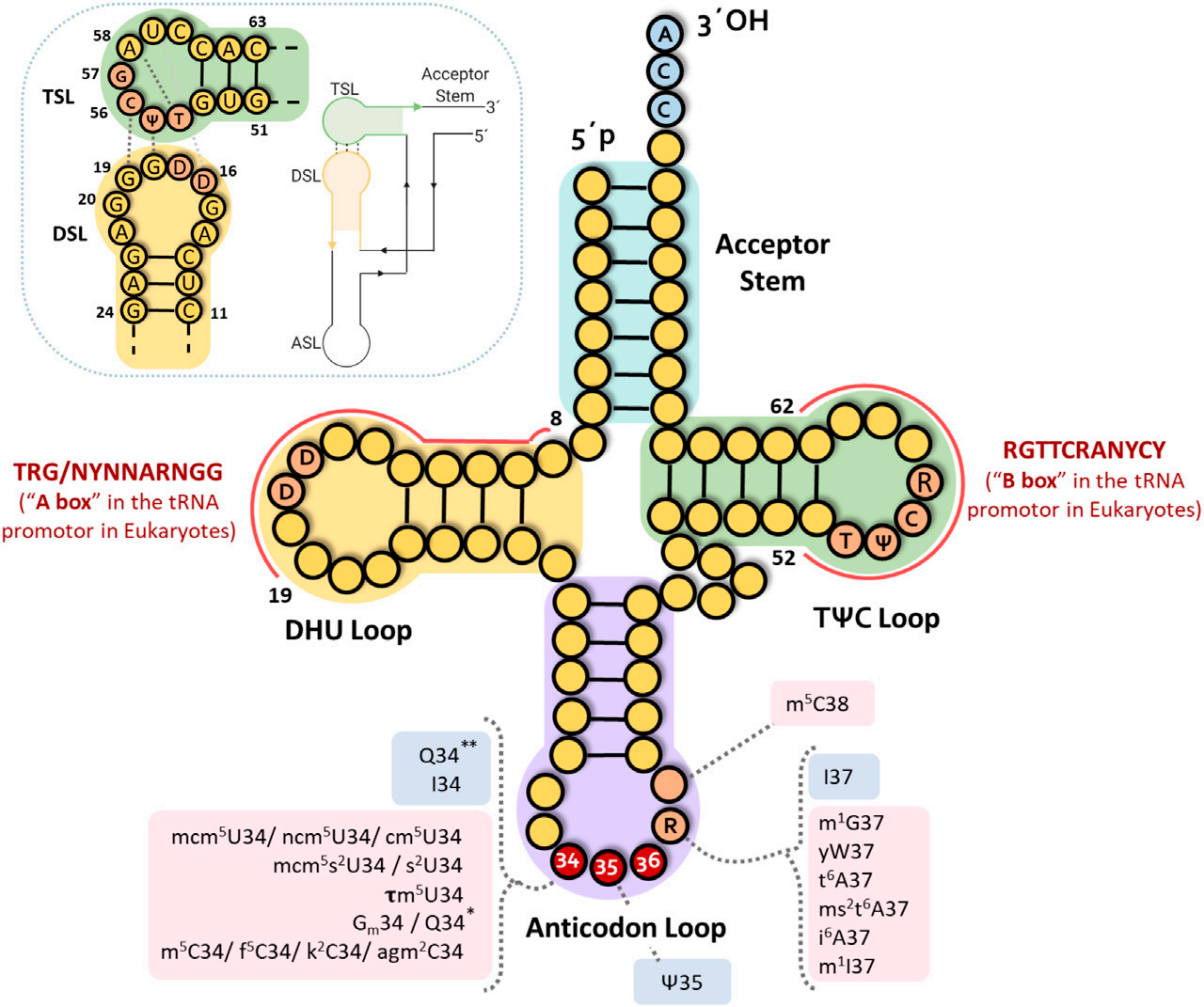
tRNA structure. Classical clover leaf fold presenting the standard base numbering. DHU and TѰC(R) loop domains corresponding to A and B boxes in eukaryotic tRNA genes are indicated. Principal interactions for the L-shape formation are shown in the upper left corner. Most frequent modified nucleosides in the tRNA anticodon are indicated. Derivatives of adenosine: t^6^A (N6-threonylcarbamoyladenosie), ms^2^t^6^A (2-methylthio-N6-threonylcarbamoyladenosine), ms^2^i^6^A (2-methylthio-N6-isopentenyladenosine), I (inosine), m^1^I (1-methylinosine). Derivatives of cytidine: Cm (2′-O-methylcytidine), m^5^C (5-methylcytidine), m^3^C (3-methylcytidine), f^5^C (5 formylcytidine), k^2^C (lysidine), agm^2^C (agmatinylcytidine). Derivatives of guanosine: Gm (2′-O-methylguanosine), m^1^G (1-methylguanosine), Q* and Q** (stand for queuosine in prokaryotes and eukaryotes, respectively), yW (wybutosine). Derivatives of uridine: cm^5^U (5-carboxymethyluridine), ncm^5^U (5-carbamoylmethyluridine), mcm^5^U (5-methoxycarbonylmethyluridine), mcm^5^s^2^U (5-methoxycarbonylmethyl-2-thiouridine), mcm^5^Um (5-methoxycarbonylmethyluridine), Ѱ (pseudouridine), s^2^U (2-thioruridine), tm^5^U (5-taurinomethyluridine) (see https://iimcb.genesilico.pl/modomics/) (Boccaletto et al., 2018). Pink indicates anticodon loop base modifications introduced by enzymes requiring cofactors and/or molecules involved in metabolic pathways. Blue boxes indicate modifications introduced by enzymes that do not require any cofactor.

To deeper understand the significance of the absent genes, we will first present a summary of missing tRNA genes for bacteria, archaea, and eukaryotes, as “anticodon charts.” Then, we will discuss the selected evolutionary options in light of the recent developments in the field, highlighting sets of excluded genes that appear linked to disambiguation needs, not only related to the recognition of the corresponding codons. These considerations bring forward the idea of further expanding the concept of adaptive molecules, in line with the growing field of non-canonical tRNA functions. In what follows, we adopt the standard numbering for tRNA bases, defining positions 34–36 as corresponding to the anticodon (see Figure 1).

## Absent Isoacceptor TRNA Genes: A General Description

**FIGURE 2:**
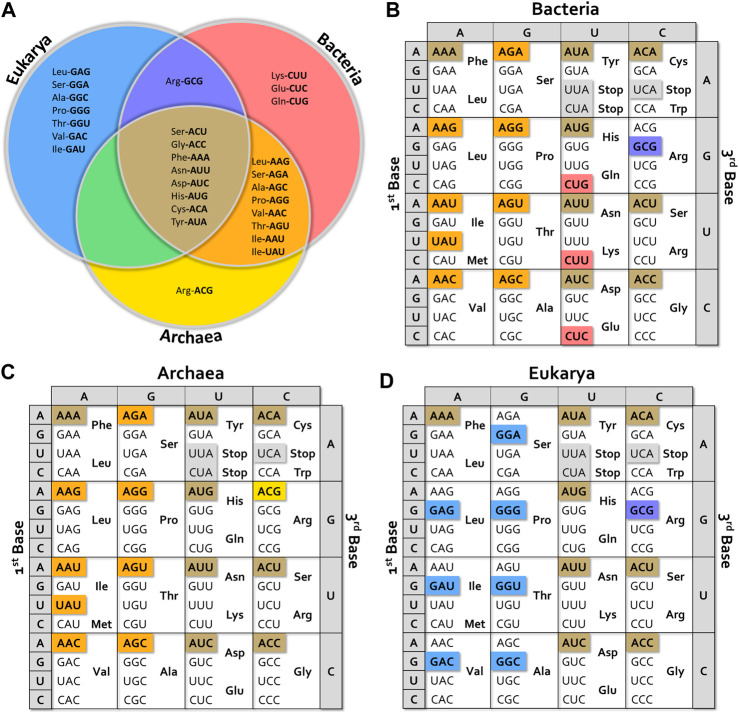
Missing tRNA anticodon alternatives. **(A)** Venn diagram showing missing tRNA isoacceptors in the different kingdoms. **(B–D)** Anticodon charts showing isoacceptors missing for bacteria, archaea and eukaryotes. The color of each box corresponds to the different sections of the Venn diagram. Tables were built considering data from more than 100 species of Bacteria (representing Acidobacteria, Actinobacteria, Aquificae, Bacteroidetes, Chlamydiae, Chlorobi, Chloroflexi, Cyanobacteria, Deinococcus-Thermus, Firmicutes, Tenericutes, Thermodesulfobacteria, and Thermotogae phyla), more than 50 species of Archaea (representing Crenarchaeota, Euryarchaeota, Korarchaeota, Nanoarchaeota, and Thaumarchaeota phyla), and more than 60 species of Eukarya (including representatives of Apicomplexa, Bryophyta, Cephalochordate, Echinodermata, Fungi, Insecta, Mammalia, Mollusca, Nematoda, Spermatophyta, and Vertebrata clades).

Absent isoacceptor tRNAs in bacteria, archaea and eukaryotes (cytoplasmic, mitochondrial and chloroplastic) are summarized as “anticodon charts” in Figure 2 and Supplementary Figure S1, that show that some isoacceptor tRNA genes are universally missing, while others are absent in particular groups. Some interesting exceptions are mentioned below.

As ***general facts*** *,* we observe that at least one isoacceptor tRNA per box is lost in all organisms. Eight tRNA genes are universally missing; tRNA^Gly^ACC and all possible ANN anticodon bearing-tRNAs in split boxes (including those with stop codons), except for tRNA^Ile^ (see Figure 2A). No UNN anticodon has disappeared with the exception of UAU in the Ile-Met box in prokaryotes.

In ***Bacteria***, the vast majority of species lack tRNA isoacceptors bearing ANN anticodons with the exception of ArgACG (instead, the ArgGCG is missing), UAU anticodon in the Ile-Met box, and CNN anticodons in His-Gln, Asn-Lys and Asp-Glu split boxes (Figure 2B). Most bacterial genomes encode a tRNA^Ile^CAU. It should be noted that the presence of genes coding for ArgACG tRNA is correlated with the existence of an adenosine-deaminase gene (*tadA*) specific for A in the first position of the anticodon (A34). Thus, the corresponding anticodon is actually ICG. It is interesting to mention some exceptions to this general description. In addition to tRNA^Arg^ACG, *Leuconostocaceae* (*Leuconostoc* and *Enococcus* sp.) carries genes for tRNA^Leu^AAG and tRNA^Thr^AGT instead of the GNN-carrying tRNAs. Some *Firmicutes* species have a gene for a tRNA^Leu^AAG and all sequenced *Thermotogae* and *Spirochaetes* contain a tRNA^Arg^GCG instead of tRNA^Arg^ACG. Interestingly, *Mycoplasma* spp. and other *Mollicutes* additionally lack the gene coding for tRNA^Arg^CCG (Yokobori et al., 2013). Data available for ***bacteriophage***-encoded tRNAs revealed that they lack the same genes absent in *bacteria* (Morgado and Vicente, 2019). Despite the very high genetic variability of phages (Hatfull, 2015; Pope et al., 2015), these tRNA genes are also considered as “prohibited,” strongly suggesting that they would be associated with viral spread drawback.

In ***Archaea*** (Figure 2C), all ANN anticodon options are missing. As in bacteria, the IleUAU is also lost.

In ***Eukarya*** (Figure 2D), one anticodon per box is lost, regardless of stop codons. These include the eight universally missing ANN anticodons and eight GNN anticodons that correspond to the eight boxes where A34 can be deaminated to inosine, namely the seven degenerated boxes except Gly, plus the Ile-Met box. Among the few exceptions to this general scheme, it is worth mentioning that *Saccharomyces* spp. and *Candida glabrata* have a gene coding for tRNA^Leu^GAG, while no genes coding for tRNA^Leu^AAG were identified. Interestingly, although the tRNA^Leu^GAG is transcribed in *S. cerevisiae*, its deletion did not impair yeast growth. On the other hand, deletion of tRNA^Leu^UAG was lethal (Huang et al., 2012). This raises questions about the cellular role of tRNA^Leu^GAG, for which no conclusive evidence about its aminoacylation is available. Additionally, this also highlights the issue of decoding LeuCUC codon, for which a superwobble mechanism has been proposed (Huang et al., 2012).

The ***mitochondrial*** tRNA population can be either entirely or partially encoded by the mitochondrial genome or be fully encoded by the nuclear genome (Schneider, 2011; Salinas-Giegé et al., 2015). In the last two cases, a highly specific mitochondrial import process is required to complete the tRNA set (Duchêne et al., 2009; Schneider, 2011; Salinas-Giegé et al., 2015). In any of those contexts, the mitochondrial tRNA population (http://trnadb.bioinf.uni-leipzig.de/ and http://plantrna.ibmp.cnrs.fr/) shows similar traits: 1) only one anticodon is conserved in fully degenerated boxes, being UNN the most commonly used and few exceptions are observed (ArgACG in mitochondria of Cestoda and in some plants, and ThrGGU and GlyGCC in some plants); 2) in split boxes, the sole options are GNN and UNN; 3) Ile and Trp tRNA coding genes present different alternatives: in plants, tRNA Ile anticodons are GAU or CAU, while in animals only option GAU is present; concerning Trp anticodon, the options are CCA in plants, UCA in animals and in *Saccharomyces* (see Supplementary Figure S1A).

It is interesting to recall that plant mitochondrial tRNA populations have been described to be mainly built through nuclear import and gene transfer processes (Maréchal-Drouard et al., 1990; Dietrich et al., 1996; Knie et al., 2015), in particular from other plants, algae, chloroplasts, fungi and bacteria (reviewed in Warren and Sloan, 2020). This complex origin and great diversity underscore the significance of the constraints that have led to the selection of an almost universal set of mitochondrial isodecoder tRNA anticodons with only few variants.

Sequence information about ***chloroplast*** tRNA is still scarce, hence their frequency cannot be precisely estimated (less than forty entries in databases: PlantRNA database (http://plantrna.ibmp.cnrs.fr/, http://trnadb.bioinf.uni-leipzig.de/) (Michaud et al., 2011; Cognat et al., 2013). However, it is possible to extract a clear general profile for angiosperms, with only small variants in bryophytes, algae and diatom plastids (see Supplementary Figure S1B). In a general way, the selected options present common traits with those of mitochondria. G/UNN anticodons have been selected in almost all boxes, except for Leu and Ala where GNN is also absent, and for Arg in the degenerated box where only ACG is present. It should be mentioned that an eukaryotic adenosine deaminase (ADAT) specific for A34 in tRNA^Arg^ACG has been identified in chloroplasts (Karcher and Bock, 2009).

## Possible Clues for the Absence of tRNA Genes

As central adaptor molecules in translation, tRNA must ensure the specificity of the interactions with different macromolecules and macromolecular complexes. The shape, conformational properties and optimal steric positioning of chemical groups at the base edges, constitute key elements to ensure the fidelity of the mechanisms in which tRNAs are involved. Very particularly, they have to be recognized by the corresponding aminoacyl-tRNA synthetase and loaded with the correct amino acid. In addition, they must be localized into the ribosome by specific factors and recognize the corresponding mRNA triplets at the ribosome decoding site. Proper accommodation is crucial since tRNAs also contribute to the concerted conformational changes that relocate the aminoacylated tRNA in the A site, dissociation of the corresponding elongation factor, and triggering of the peptidyl-transferase activity. Moreover, they play a role in the concerted movement that leads to the release from A site. Given such vital functions, the absence of particular tRNAs appears as a striking observation. What could be the causes that explain the systematic exclusion of certain tRNA genes? Some possible clues are addressed in the following sections.

The present analysis was centered on the tRNA populations defined only by their anticodon, though other structural elements and several supplementary levels of adjustment are required to ensure a faithful and efficient translation (for a general review see Agris et al., 2018). Although the anticodon triplet clearly defines the tRNA identity referred to the cognate amino acid and codon recognition, specific interactions in the decoding site further involve the overall anticodon-stem and loop (ASL). In particular, neighboring bases stabilize the codon-anticodon interaction, prevent slippage and frame-shifting, and increase decoding fidelity. Within critical ASL elements, much work has been focused on the role of the 32–38 base-pairing, and the conserved and frequently modified or hypermodified purine at position 37, among others (Konevega et al., 2004; Olejniczak and Uhlenbeck, 2006; Ledoux et al., 2009; Pernod et al., 2020). Interestingly, structural studies showed that initial binding of tRNA to the A site is followed by a rate-limiting rearrangement of the anticodon loop in the ribosome decoding center that is favored by the purine 37, yielding additional interactions with the rRNA (Konevega et al., 2004).

### About Database Curation, Exceptions and Exclusions

The systematic absence of particular tRNA genes, related to alternatives in the first anticodon base (base 34, the “wobble position”), suggests that those anticodons would be considered as “deleterious” and then might be cleared off by negative selection. This is an interesting phenomenon to underline, considering the high number of copies and variants of tRNA gene isotypes, especially in eukaryotes. Indeed, a considerable number of putative tRNA coding sequences carrying “prohibited” anticodons could be found in eukaryotic genomes in earlier genomic tRNA databases. Most of them have been eliminated in the last release of the GtRNAdb (http://gtrnadb.ucsc.edu/ data release 18:1, August 2019) based on high stringency algorithms. Even though some tRNAs are still signaled as functional tRNAs, they contain substitutions in other regions (mainly at the level of the TѰCR and DHU loops), that would affect the stability of the “L-shape” and/or the helical fold and stability of different arms (in particular mispairings and/or higher number of G-U base pairs). Except for a few recently reported cases discussed below, there is no information yet about the transcription of most of these sequences. Moreover, in eukaryotes, modifications of the genomic sequences encoding the DHU and TѰCR arms and loops also affect the A and B sites of the tRNA gene promoters, altering their transcription (see Figure 1). However, if these sequences are indeed transcribed, they would fold into a non-canonical structure inconsistent with the ribosomal-dependent translation function.

### The Principle of the Excluded Purine: A Matter of Three Purines

Purine selection in the first anticodon position appears as one of the major elements in structuring the extant genetic code. Isoacceptors containing A or G are mutually excluded throughout the phylogeny. This was already highlighted by different authors as one of the major anticodon-sparing strategies (Marck and Grosjean, 2002; Maraia and Arimbasseri, 2017). In addition, the non-canonical inosine nucleoside (product of adenosine deamination) appears as a third option and its role has been recently underlined (Torres et al., 2014b; Rafels-Ybern et al., 2018). It is found in all domains of life at three possible positions on tRNAs: 34 (the wobble position), except for archaea; 37 (following the anticodon); and 57 (at the TѰCR loop). As early proposed by Crick in the wobble hypothesis (Crick, 1966), inosine at the wobble position has an obvious implication on codon-anticodon recognition, since it is capable of pairing with A, U and C, while A34 only pairs with U. Furthermore, it was suggested that I34 introduction might play a major role in driving codon usage-biased translation to shape proteome landscape (Rafels-Ybern et al., 2019).

Adenosine in the first position of the anticodon appears throughout the phylogeny linked to the presence of A34 specific adenosine deaminases. Indeed, no A34-specific ADAT has been identified in archaea. In most bacteria, the only tRNA bearing adenosine at this position is ArgACG, which is in fact transformed to inosine by a homodimeric TadA. The same modification of ArgACG is introduced in chloroplasts by an ADAT imported from the cytoplasm (Delannoy et al., 2009). However, no A34 has been identified yet for mitochondrial tRNAs. In the vast majority of eukaryotes, a heterodimeric ADAT (composed of ADAT2/Tad2 and ADAT3/Tad3) modifies cytosolic tRNA species with ANN anticodons (tRNA^Arg^ACG, tRNA^Ala^AGC, tRNA^Ile^AAU, tRNA^Leu^AAG, tRNA^Pro^AGG, tRNA^Ser^AGA, tRNA^Thr^AGU and tRNA^Val^AAC) (Torres et al., 2014b; Rafels-Ybern et al., 2018)*.*

#### One More Step on the Purine Conflict: The Q Link Between Bacteria and Eukaryotes

An additional complexity level in the purine conflict is introduced by the replacement of guanosine in Tyr, His, Asn and Asp tRNAs (all found in split boxes) with the hypermodified queuine base (Q) at the position 34 of tRNA anticodons. Queuine is a 7-deaza-guanosine derivative, synthesized by eubacteria and salvaged by eukaryotes for its incorporation into tRNA (Fergus et al., 2015; Müller et al., 2019). In higher eukaryotes, queuine is further modified in tRNA^Tyr^ and tRNA^Asp^ by the addition of a galactose and a mannose sugar, respectively (Fergus et al., 2015). The base-exchange reaction resulting in Q incorporation is carried out by the tRNA guanine transglycosylase (TGT). Albeit TGTs are present in all kingdoms, conserve structural traits, and share the catalytic mechanism, archaea TGT do not modify the first anticodon position and instead incorporate a queuine analogue, archaeosine, at position 15 in the D-loop of many tRNAs (Phillips and de Crécy-Lagard, 2011; Turner et al., 2020).

#### The Purine Conflict: An Information or a Structural Problem?

Interestingly, in all extant organisms, A and G are mutually excluded at the first position of the anticodon within tRNAs carrying the same amino acid (except tRNA^Ser^ in eukaryotes). This means that tRNAs isoacceptors bearing the other purine at this position are somehow excluded. In prokaryotes, guanosine is the only purine used at the wobble position, except for tRNA^Arg^ in bacteria. In eukaryotes instead, the choice of the purine at this position is diverse. Eukaryotes use eight tRNAs with inosine (seven in the cases of *Saccharomyces* spp. and *C. glabrata* mentioned in previous sections), four have queuine and four harbor guanosine. The four eukaryotic tRNAs maintaining guanosine at position 34 are: PheGAA, GlyGCC, CysGCA and SerGCU (in the split box shared with Arg). In organisms in which the tRNA sequences were determined, the corresponding G34 appears to be modified in the following cases (http://trnadb.bioinf.uni-leipzig.de/): one of the two tRNA^Gly^GCC reported for *S. cerevisiae*, and all tRNA^Phe^GAA (a 2MeO modification of G34 is almost universally present, except an unknown G modification reported for *Mus musculus*, and the absence of modifications in *Mycoplasma capricolum*, *Rhodospirillum rubrum* and archaea)*.* So far, no modifications of G34 have been described for tRNA^Cys^GCA and tRNA^Ser^GCU. All these data suggest that the choice of the purine at position 34 is critical, as confirmed by a recent report studying the use and putative toxicity of G34 in eukaryotes (Pernod et al., 2020).

What could be the physical basis for the purine conflict that leads to the exclusion of one of the three options? Undoubtedly, interaction alternatives allowed by inosine in synonymous codons recognition is a major element. However, this may not be the only cause if bacteria and archaea tRNAs are considered, as this modification is only present in tRNA^Arg^ACG. Most tRNA modifications are directly linked to the metabolic state through the requirement of key cofactor molecules, from which S-adenosylmethionine as chemical group donor and thiol source is the main paradigm (Helm and Alfonzo, 2014; Danchin et al., 2020). However, adenosine deamination, Q/G transglycosylation (in eukaryotes) and pseudouridylation (that also involves a transglycosylation mechanism) (Veerareddygari et al., 2016) are not directly related to the metabolic state of the cell. Hence, the purine conflict should be associated with other general common constraints.

An attractive alternative would be to consider the shape of tRNA accepted by the sites in the ribosome, and the specific interactions that take place there and that are mostly conserved in all kingdoms of life (Fox, 2010; Melnikov et al., 2012; Petrov et al., 2014). Another critical factor would be the role of different tautomeric and protonation properties and states of the three purines in defining ambiguity levels admitted in the recognition process in the ribosome. Nucleic acid bases can adopt multiple tautomeric forms due to the presence of multiple solvent-exchangeable protons (reviewed in Shukla and Leszczynski, 2013; Singh et al., 2015) and this plays a key role in the specificity of the interactions in which they are involved (Westhof, 2014; Agris et al., 2018).

Crystallographic studies showed that the ribosomal grip around the triplet codon/anticodon fits sterically better with the dimensions and volume of a standard RNA helix that is recognized through the shallow minor groove (Murphy and Ramakrishnan, 2004; Demeshkina et al., 2012). This helix may contain Watson–Crick-like pairs involving particularly-protonated bases, tautomeric and modification states, as well as *anti-syn* conformation options, resulting in different H-bonding alternatives at their edges (Westhof et al., 2014). There is now extensive structural information about the codon/anticodon interaction in the ribosome showing the critical role of modified bases (Westhof, 2014; Westhof et al., 2014). So far, only some evidence has been presented regarding variants of the tautomeric state of the bases in the wobble position. In addition, for I34-bearing anticodons, it was reported that while I-C base-pair stericity was similar to the canonical G-C base pair, the purine-purine I-A pairing has an increased width that requires a change in the geometry of the anticodon to fit the conserved interactions at the decoding center (Murphy and Ramakrishnan, 2004).

Additionally, it was recently communicated that the I34-A3 base-pair is tightly dependent on the anticodon loop conformation and the modification state of neighboring bases (Vangaveti et al., 2020). Alltogether, these observations highlight the role of base tautomerism in base pairing schemes related to the steric and geometric constraints imposed by the ribosome as originally discussed by Topal & Fresco (Topal and Fresco, 1976).

### Too Many Partners for a Common Language: Split Box Informative Problems

#### The Ile-Met Box Dilemma

The genetic code establishes that three codons correspond to Ile and one to Met. Their box can be considered either as originally completely degenerated with all four triplets corresponding to Ile and invaded by Met, or as split box invaded by Ile leaving only one option for initiator or elongator Met (Lei and Burton, 2020). Notably, functional tRNAs in this box are different in eukaryotes and prokaryotes.

In the former, the anticodon GAU is missing, AAU yields IAU, and UAU anticodon is modified to ѰAU, ensuring the recognition of AUC, AUU and AUA codons. In prokaryotes, however, AAU and UAU anticodons are lost, and CAU anticodon is shared by tRNAs for Ile and Met. Nonetheless, tRNA^Ile^CAU and tRNA^Met^CAU are perfectly discriminated by the corresponding synthetases, and specifically recognize Ile and Met codons, respectively. The mechanism ensuring discrimination is different in bacteria and archaea. In both cases, a post-transcriptional modification of cytosine takes place in the anticodon of the tRNA^Ile^CAU (Suzuki and Numata, 2014). In almost all bacteria, C34 is modified with a lysidine (k^2^C) residue catalyzed by a tRNA^Ile^ lysidine synthetase (TilS) (Soma et al., 2003). In archaea, C34 in IleCAU anticodon is modified with agmatine, yielding 2-agmatinylcytidine (agm^2^C) (Mandal et al., 2010). Agmatine is an intermediate metabolite in the polyamines pathway, generated from arginine via decarboxylation by the enzyme arginine decarboxylase (TiaS).

Conjugation of lysine or agmatine to the C2 carbon of cytosine by deoxidization induces a tautomeric change of cytosine from enamine to imine, with eventual protonation of N3. Thus, these modifications completely alter the proton donor-acceptor pattern of cytosine, preventing base-pairing with G and enabling that with A (Voorhees et al., 2013), avoiding MetAUG codon misreading. Interestingly, TilS and TiaS enzymes belong to non-related protein families that modify the wobble cytidine by distinct catalytic mechanisms: TilS activates the C2 carbon by adenylation and TiaS activates it by phosphorylation (Numata, 2015). However, their activity has the same physical and physiological effect. Hence, this constitutes an interesting case of convergent evolution since the decoding system for AUA codons would have appeared after the separation of bacteria and archaea from their common ancestor (Suzuki and Numata, 2014). In addition, it is a notable case of storing different information in the anticodon of the tRNA, which allows, on one hand, disambiguation to read the mRNA and, on the other, proper recognition and acylation by the cognate aminoacyl-tRNA synthetase.

#### Charging Amide Amino Acids in Prokaryotes

Isodecoder tRNAs selected within NUN split boxes differ between bacteria and archaea. As in general cases, ANN anticodons have not been retained. In addition, CUN anticodon is also missing in bacteria, yielding a single decoder tRNA species for Gln, Asn, Lys, Asp and Glu amino acids. Interestingly, most bacterial and all known archaeal genomes do not encode glutaminyl-tRNA synthetase (GlnRS) and a large number of prokaryotes do not have an asparaginyl-tRNA synthetase (AsnRS) (Tumbula et al., 2000; Sheppard and Söll, 2008; Nureki et al., 2010) even though they have a tRNA^Gln^UUG and a tRNA^Asn^GUU. In those cases, charging the corresponding amino acid on the cognate tRNA occurs by an indirect pathway: the tRNA is first charged with Glu or Asp by glutamyl-tRNA synthetase (GluRS) or aspartyl-tRNA synthetase (AspRS) respectively, and then amidated by specific amidotransferases (Sheppard et al., 2008). There are two tRNA-dependent amidotransferases (AdT) described: the heterotrimeric GatCAB in both archaea and bacteria, and the heterodimeric GatDE only in archaea missing AsnRS. GatCAB is required to catalyze the conversion of Glu-tRNA^Gln^ and/or Asp-tRNA^Asn^ into Gln-tRNA^Gln^ and/or Asn-tRNA^Asn^. In a similar way, GatDE is involved in the Gln-tRNA^Gln^ formation (for a review see Sheppard and Söll, 2008; Sheppard et al., 2008). Therefore, AdTs must discriminate their mischarged tRNA substrates from the cognate aa-tRNA species. Interestingly, the co-crystal structure of GatDE with tRNA^Gln^ in *M. thermautotrophicus* showed that AdTs would achieve this discrimination without directly recognizing the anticodon of their tRNA substrates (Oshikane et al., 2006), suggesting a possible involvement of different constraints dispersed in the tRNA structure.

The two-step process to introduce amide amino acids raises complex evolutionary questions while providing interesting clues (Sheppard and Söll, 2008; Di Giulio, 2020). On the one hand, they have been associated with the general metabolic roles of these amino acids in various prokaryotes (Sheppard and Söll, 2008). On the other, they must ensure translation fidelity in cases of anticodon pairs UUC/UUG (Glu/Gln) and GUC/GUU (Asp/Asn) where a supplementary discrimination step is required, complementing the codon-anticodon recognition.

The post-aminoacylation amidation mechanism opens up the possibility that unmodified Asp-tRNA^Asn^ and Glu-tRNA^Gln^ can reach the decoding site and introduce an error. However, it has been demonstrated that the elongation factor EF-Tu discriminates misacylated from correctly amidated tRNA. This not only sheds light on an additional mechanism for ensuring translation fidelity, but also reveals that the elongation factor constitutes a further partner to read the selected coding system (Roy et al., 2007; Eargle et al., 2008).

#### Cys-Trp-Stop, Tyr-Stop, Phe-Leu and Ser-Arg Split Boxes

In the Cys-Trp box, the anticodon option CysACA is universally absent, and there is a single option for Cys and for Trp. The fourth triplet, corresponding to the opal termination codon (UGA) can be decoded by the rare tRNA^Sec^UCA carrying selenocysteine, the infrequent 21st amino acid. Selenocysteine does not have a dedicated synthetase in any of the three kingdoms. Sec-tRNA^Sec^ is synthesized by the conversion of serine through a multistep process in a Sec-specific tRNA-dependent manner. tRNA^Sec^ is first aminoacylated with Ser by seryl-tRNA synthetase (SerRS) to produce Ser-tRNA^Sec^. The following step is species-dependent: in bacteria, Sec synthetase (SelA) converts Ser-tRNA^Sec^ to Sec-tRNA^Sec^ in a single-step reaction; in contrast, archaea and eukaryotes carry on the synthesis through an intermediate step where the Ser moiety is first phosphorylated (Yuan et al., 2010; Wang et al., 2015; Holman et al., 2017).

In a number of methanogenic archaea, a cysteinyl-tRNA synthetase (CysRS) is either absent or dispensable. In those cases there is a tRNA-dependent indirect pathway in two steps. The tRNA^Cys^ is initially aminoacylated with O-phosphoserine (Sep) by O-phosphoseryl-tRNA synthetase (SepRS). The Sep moiety is subsequently transformed to a tRNA-bound cysteine by Sep-tRNA:Cys-tRNA synthetase (SepCysS) (Sauerwald et al., 2005). Finally, concerning this box, it should be reminded that in animals and *Saccharomyces* mitochondria, the Trp anticodon is UCA and UAG, respectively, and that they universally correspond to opal and amber stop codons.

In the Tyr box, the AUA option is universally absent, GUA is modified to QUA in bacteria and eukarya, and there are two stop codons: the amber UAG and the ochre UAA. The amber codon is read by a rare tRNA^Pyr^CUA, up to now only described in *Methanosarcinaceae* archaea and in bacterial phyla *Clostridia* and δ*-proteobacteria.* This tRNA is charged with pyrrolysine - the uncommon 22nd amino acid—by a dedicated pyrrolysine-tRNA synthetase (Yuan et al., 2010; Wan et al., 2014).

In the Phe-Leu and Ser-Arg split boxes, the option A34 is universally missing, leaving a 1-2 distribution for the two amino acids.

As briefly summarized above, the information available on the tRNAs corresponding to split boxes, strongly suggests that many constraints and options have accompanied the evolution of the translational apparatus ensuring its fidelity, and are condensed in their structures as a kind of “molecular algorithm.”

### The Mystery of tRNA Gly Anticodons

The intriguing absence of an A in the wobble position of Gly degenerated box in all organisms has already been a particular focus of attention (Novoa et al., 2012; Maraia and Arimbasseri, 2017). It is interesting to note that the human isoacceptor tRNA^Gly^GCC, with its anticodon experimentally changed to either ACC or ICC, was shown to be recognized and loaded with Gly by glycyl-tRNA synthetase (GlyRS) (Saint-Léger et al., 2016). This strongly suggests that in this case, the purine at position 34 would not play a critical role in the discrimination by the cognate synthetase. Furthermore, they showed that the anticodon loop of the different chimeric tRNA^Gly^ did not interfere with binding but prevented deamination by hADAT. Complementary molecular dynamics studies showed that the introduction of an A34 destabilized the structure of tRNA^Gly^ anticodon. This led to propose the challenging hypothesis that a kind of “signal saturation” would have been reached limiting the evolution of extant genomic populations of tRNAs (Saint-Léger et al., 2016).

It is interesting to link these observations with the idea that tRNA^Gly^ would be the first tRNA, possibly initially loaded by a ribozyme and that GlyRS would be the primordial aminoacyl-tRNA synthetase (Lei and Burton, 2020).

### Diacritic Marks in tRNA Languages: Disambiguation and Link With Cellular State

Giegé et al. (2012) have described the modified bases of tRNA as true diacritical marks. The critical role of modified bases of ribonucleic acids, very particularly those of tRNAs, have been recognized since the early work of Grosjean and Björk (Björk and Neidhardt, 1975; Grosjean et al., 1995, Grosjean et al., 2010; Grosjean, 2015). Nowadays, they have become the subject of massive work in light of the large diversity of functions in which these molecules are involved (Phizicky and Hopper, 2015; Tuorto and Lyko, 2016; Agris et al., 2017; Marín et al., 2017; Barraud and Tisné, 2019). Particularly, there is an increasing association between the modified status of tRNAs and a number of pathologies (Torres et al., 2014a; Huang et al., 2018; Pereira et al., 2018; Santos et al., 2019). Within the scope of this review, we would like to emphasize that the enzymes involved in a large number of post-transcriptional modifications of tRNA (methylation, thiolation and incorporation of amino acid derivatives, dihydrouridine synthase, among others) require cofactors that constitute key molecules in metabolic pathways. Within them, it is worth mentioning S-adenosylmethionine (AdoMet), folate and NADPH (see Figure 1) (Helm and Alfonzo, 2014; Danchin et al., 2020).

The vast majority of the modifications in the base at position 34 and in the corresponding ribose (excluding I and Q in eukaryotes), very particularly U34, as well as in the neighboring bases of the anticodon, require the participation of some of the above-mentioned cofactors. It seems relevant to highlight the high metabolic cost of modifying U34, that relies on several enzymatic steps including methylation and thiolation processes (El Yacoubi et al., 2012; Karlsborn et al., 2014; Ranjan and Rodnina, 2016; Boccaletto et al., 2018), or the biosynthesis of the modified guanine Yw base, which may require up to six AdoMet molecules (Young and Bandarian, 2013). All this indicates the close link between the state of these tRNA modifications and the cellular metabolic context. On the contrary, deamination of A34 in bacteria and eukaryotes, and introduction of Q34 in eukaryotes, seem to be essentially linked to disambiguation requirements in translation. In bacteria, queuosine biosynthesis is an expensive metabolic process, requiring AdoMet, THF, NADPH and GTP (Slany and Kersten, 1994; Phillips et al., 2008).

It is interesting to feature the close link between the set of processes involved in one-carbon metabolism with the translation apparatus through tRNA modifications, that has been nicely reviewed elsewhere (Helm and Alfonzo, 2014; Danchin et al., 2020).

### Missing tRNAs or Large Reserves of Functional Alternatives?

The role of the high number of tRNA genes is still unclear (Iben and Maraia, 2014; Maraia and Arimbasseri, 2017; Pan, 2018; Torres, 2019) and it was suggested that an important part of them are actually silent (Torres, 2019). Interestingly, given the large size of the cellular tRNA population (which in higher eukaryotes is estimated to be around tens of millions of copies), it is expected that they participate in a large number of interactions with other macromolecules with a high degree of specificity in the context of several cellular processes besides protein synthesis (Pan, 2018). Half a century has passed since the first reports on extra-ribosomal roles of tRNAs (Soffer et al., 1969; Leibowitz and Soffer, 1970), and many evidence has accumulated on the so-called “non-canonical” functions of tRNAs to differentiate them from their key role in ribosomal protein biosynthesis (Raina and Ibba, 2014; Katz et al., 2016; Schimmel, 2018; Su et al., 2020). The list continues to grow, most particularly with the increasing description of cellular properties of tRNA fragments that have received much attention during the last years. All this leads to think about the significance of the tRNA population in the cell in a whole new way. Therefore, it is appealing to establish a correlation between the high number of putative tRNA gene sequences coding for tRNAs with non-canonical structures and the diversity of functions described. This poses semantic problems referred to the nomenclature of the genes, and still logistical problems linked to the search and identification of their possible products.

**FIGURE 3:**
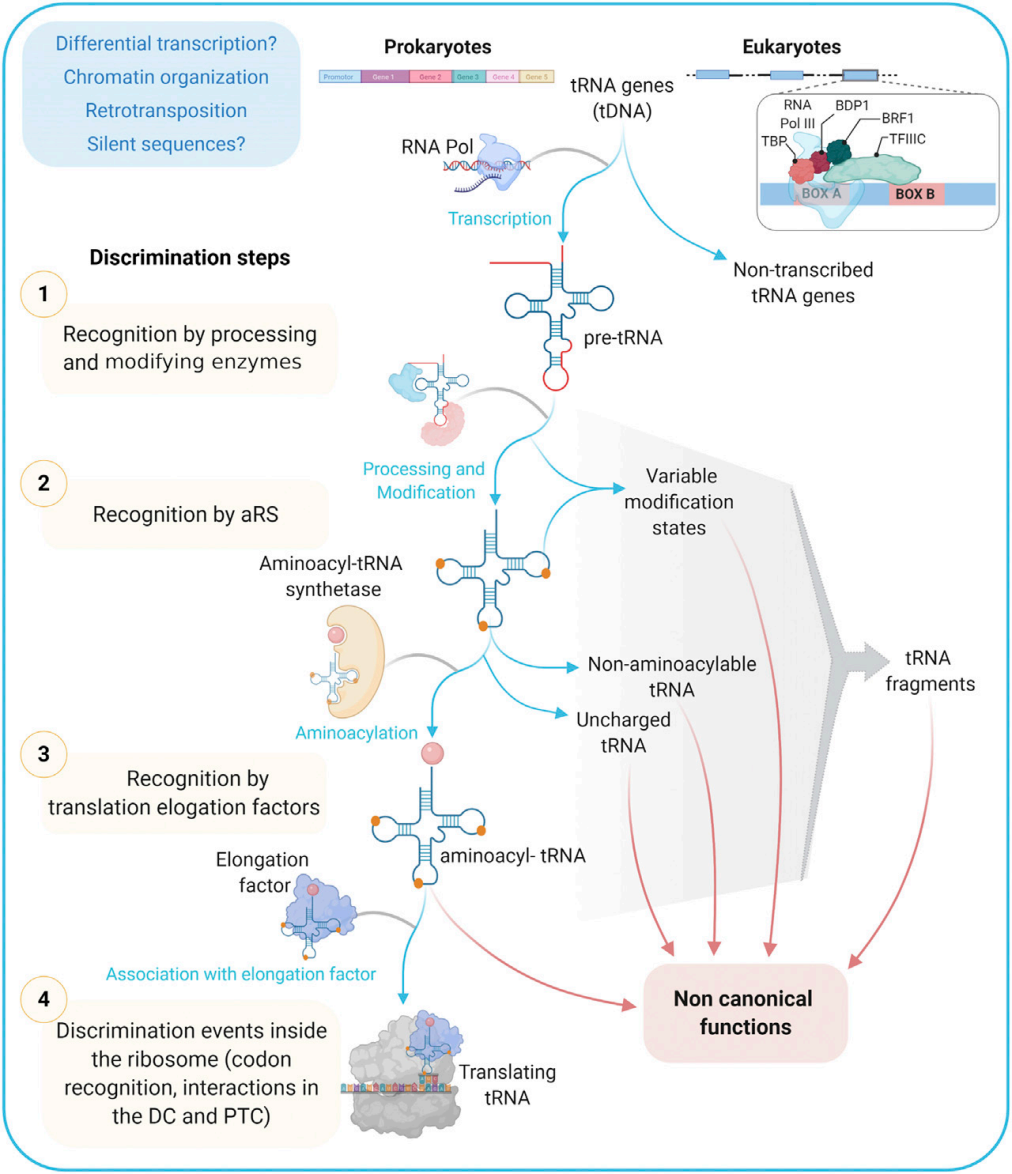
tRNA informative layers. From transcription to codon recognition in the ribosome, tRNAs should go through many discrimination steps to ensure the translation fidelity. Alternative paths from these steps/checkpoints may provide a wide repertoire of tRNAs with functions different to the ribosomal protein synthesis and that are together referred to as non-canonical ones. *TBP:* TATA Binding Protein, Transcription Factor (TF); *BDP1:* B Double Prime 1, TFIIIB subunit also known as TFIIIB150; *BRF1:* TFIIIB 90 kDa subunit, *TFIIIC:* Transcription Factor IIIC; *aRS:* Aminoacyl tRNA Synthetase; *DC:* Decoding Center; *PTC:* Peptidyl-Transferase Center.

A primary classification into canonical and non-canonical tRNA genes can be attempted based on the structure of both the transcript and the promoter. Canonical genes code for molecules recognized and loaded by the cognate aminoacyl-tRNA synthetase and involved in ribosomal biosynthesis of proteins. Instead, non-canonical genes, apart from the obvious non-transcribed sequences (see further below), would include: 1) sequences coding for molecules with high scores of structure conservation that are aminoacylated by the cognate synthetase, but that will contribute to non-ribosomal synthesis, 2) genes coding for molecules capable of adopting alternative folds that fulfill protein synthesis-unrelated functions (see Figure 3).

Among tRNA roles outside ribosomal protein synthesis, the following have been previously highlighted: nutrient sensing, transcription regulation, retroelement insertion, translation kinetics and protein folding, stress response, immune response, apoptosis inhibition, peptidic antibiotic biosynthesis, bacterial wall biosynthesis, post-translational protein modification, membrane lipid modification, retroviral replication, mitochondrial ribosome assembly, and mitochondrial DNA replication (Seligmann, 2010; Raina and Ibba, 2014; Katz et al., 2016; Balasubramaniam et al., 2017; Bogenhagen et al., 2018; Su et al., 2020; and this issue). Also, several roles have been described for tRNA fragments, among which the following stand out: gene silencing, translation regulation, transposable element regulation, noncoding RNA regulation, cell differentiation, cell proliferation and cancer, host defense, stress response, apoptosis, and epigenetic inheritance (Magee and Rigoutsos, 2020; Polacek and Ivanov, 2020; Su et al., 2020; and this issue). In most cases, the information about the precise isotype, state of post-transcriptional modification, recognition by the cognate aminoacyl-tRNA synthetase, and aminoacylation state is still scarce, leaving the door open for new research avenues.

Here we describe a few illustrative examples of non-canonical functions for which many layers of evidence exist. In the available *Staphylococcus aureus* sp. genomes, seven different genes have been annotated to encode tRNA^Gly^ isoacceptors. One of them, a tRNA^Gly^UCC, has a very low score in the tRNA database and presents variations in the DHU and TѰCR loops. Notably, though it is efficiently charged by the cognate glycyl-tRNA synthetase, it is not efficiently recognized by the elongation factor EF-Tu and is rather involved in the pentaglycine bridge synthesis during the bacterial wall formation (Giannouli et al., 2009). Interestingly, two other tRNA^Gly^UCC putative genes carrying similar variants in the TѰCR loop exist in the *S*. *aureus* genome.

In humans, a low-expressed and non-aminoacylable tRNA^Asp^GUC was reported to play a particular role in the regulation of aspartyl-tRNA synthetase gene transcription through direct binding to an Alu sequence in the 3′UTR (Rudinger-Thirion et al., 2011). This molecule is similar to other human tRNA^Asp^ sequences competent to adopt the classical cloverleaf tRNA structure, although it has significant changes in the TѰCR stem and loop domain. Furthermore, the authors also demonstrated that it is not able to fold into a canonical tertiary tRNA structure.

More recently, by analyzing human tRNA-Seq datasets through a bioinformatics strategy, it was shown that changes in tRNA gene expression not only include the relative abundance of mature tRNA but also significant changes in immature tRNA sequences and tRNA fragments. Importantly, this has been associated with different biological functions unrelated to protein synthesis (Torres, 2019). This approach paves the way to delve into the analysis of the biological significance of non-canonical tRNA sequences. Such non-canonical tRNAs are usually encoded by tDNAs that have been mostly (but not completely) removed from the last databases due to the use of high-stringency algorithms, mainly based on the occurrence of alterations in DHU and TѰCR loops and/or mispairings. This is exemplified by tRNA^Ala^AGC putative coding sequences in human and *Danio rerio* genomes, which dropped from 29 and 92 in 2015 to 26 and 6 in 2019, respectively. Nevertheless, sequences with variations in the TѰCR loop still remain. This strongly suggests the importance of keeping the discussion open.

The above-described cases correspond to tRNA genes carrying anticodons that are not absent, i.e., that there are other isodecoders for that particular anticodon able to perform the canonical adaptor function. Nevertheless, altogether, this data emphasizes that although several non-canonical tRNA gene sequences previously labeled as “missing genes” would be effectively silent, many others might have important activities, either similar to those described here, or even alternative functions that wait to be discovered (Pan, 2018; Torres, 2019).

### tDNA Genes and Pseudogenes: Adapter Roles in Chromatin Structure?

Unlike prokaryotes, where tRNAs are clustered in polycistronic genes and/or included in the rDNA locus, eukaryotic tRNA genes are largely monocistronic and are considered as independent units scattered throughout the genome. They constitute real middle repetitive DNA elements and part of them could be considered as pseudogenes, either silent or poorly expressed (Torres, 2019). However, they could have soundly impacts from a structural perspective.

Indeed, there is growing evidence underscoring the role of tDNAs in chromatin organization, independent of their capacity to be transcribed or not. tRNAs are transcribed by a dedicated RNA polymerase (RNA pol III) and their promoters are bound by several specific factors, including TFIIIB, TFIIIA and TFIIIC. The latter is a multi-subunit complex which recognizes the two conserved A and B elements located in the transcribed sequence overlapping the DHU and TѰCG tRNA loops respectively (Arimbasseri and Maraia, 2016; Arimbasseri, 2018) (see Figure 1). In particular, the critical binding of TFIIIC to B box sequences, both in active promoters and in scattered sequences in the genome, has been pointed out as an essential contributor to chromatin architecture (Noma et al., 2006; Wallrath and Geyer, 2006). Furthermore, it has been described that tDNA domains contain binding sites for numerous chromatin-binding proteins such as cohesins and condensins complexes, as well as some SMC (structural maintenance of chromatin) proteins (Hamdani et al., 2019) that are involved in the organization of high order chromosomes, as well as in the structure of chromatin that changes the accessibility of DNA to regulatory factors, thus impacting on gene expression (Hübner and Spector, 2010). Moreover, tDNA sequences have also been implicated in several other activities at the chromatin level. Among them stand out: nucleosome positioning, insulator role blocking the spreading of heterochromatin domains, replication fork pausing that mediates long-range interactions defining chromosome architecture, and recombination events (Noma et al., 2006; Wallrath and Geyer, 2006; Arimbasseri and Maraia, 2016; Ciesla et al., 2018; Shukla and Bhargava, 2018; Hamdani et al., 2019; Torres, 2019). Interestingly, tDNA genomic loci have also been described as targets for transposon insertion (Cheung et al., 2018).

In turn, and constituting a feed-back loop, the genomic context and the local structure of chromatin would define the bases for the differential expression of tRNA genes in eukaryotes, stressing the vital role of chromatin organization in tRNA-mediated responses (Arimbasseri and Maraia, 2016; Sagi et al., 2016; Ciesla et al., 2018; Shukla and Bhargava, 2018).

## Conclusion: Reading the Palimpsest

The challenging problem of the origin of the genetic code and the explanation of why amino acids are encoded by a different number of synonymous codons is still open (Di Giulio, 2005; Ribas de Pouplana et al., 2017; Lei and Burton, 2020) as well as it is the problem of why certain anticodons were excluded throughout evolution. Furthermore, the multiplicity of functions of these molecules adds an additional level of complexity.

There is a general conception that despite tRNAs are among the most ancient and highly conserved molecules, they are poor phylogenetic markers because they are short, and often subject to horizontal gene transfer and recombination events (Widmann et al., 2010). However, they constitute precious material for deciphering early events in the origin of life (Widmann et al., 2010; Ribas de Pouplana et al., 2017; Lei and Burton, 2020). The structure of each individual molecule and that of the entire cellular tRNA population, could be seen as a historical summary of the origin and evolution of the storage and expression of biological information, but also of the regulatory mechanisms that ensure precise adaptation between metabolism, changes of the cell program and environmental conditions.

tRNA has emerged as an adaptor molecule, associated with the first events of the establishment of life (Di Giulio, 2005). Progressively, the successful adaptor structure would have acquired new functions involving very diverse, but at the same time specific, intermolecular interactions. Without going into the possible temporal succession of evolutionary events, it is clear that the reached structure was acquiring new biological roles, both in prokaryotes and eukaryotes, accumulating signals that ensure the fidelity of the molecular interactions. This important diversity of functions fulfilled by tRNAs leads to an expansion of Crick's concept of adapter molecule (Ribas de Pouplana and Dedon, 2014).

In bacteria and archaea, the same set of tRNAs could perform canonical and non-canonical functions, as multifunctional molecules. In eukaryotes, the significant increase in the number of tRNA genes, their different genomic organization, and the existence of a dedicated RNA polymerase, would be accompanied by the appearance of specially dedicated tRNA molecules for extra-ribosomal functions, with “admitted” and “prohibited” anticodons, increasing the activities in which tRNA molecules are involved.

Canonical and non-canonical functions require the reduction of ambiguity of signals that define the specific interactions in which tRNA molecules are involved. This necessary disambiguation would be at the origin of the exclusion of particular isodecoding options. Besides the basic structural elements that ensure the fidelity of the interactions, a level of modulation of the signals deposited in the tRNA structure by post-transcriptional modifications must be also considered.

In conclusion, tRNA genes and molecules appear as a remarkable example of molecular palimpsests and evolutionary puzzles. As in the work of an archaeologist, the layers of information should be delicately removed one after the other. This advance in their decryption might now be accomplished by the use of state-of-the-art technologies.
