# Supplementary material for: On the Track of the Missing tRNA Genes: A Source of Non-Canonical Functions?
Source: Front Mol Biosci. 2021 Mar 16;8:643701. doi: 10.3389/fmolb.2021.643701 (PMC8007984; doi:10.3389/fmolb.2021.643701)
Supplement: Supplementary file 1 [file table1.docx]

***Supplementary Material***


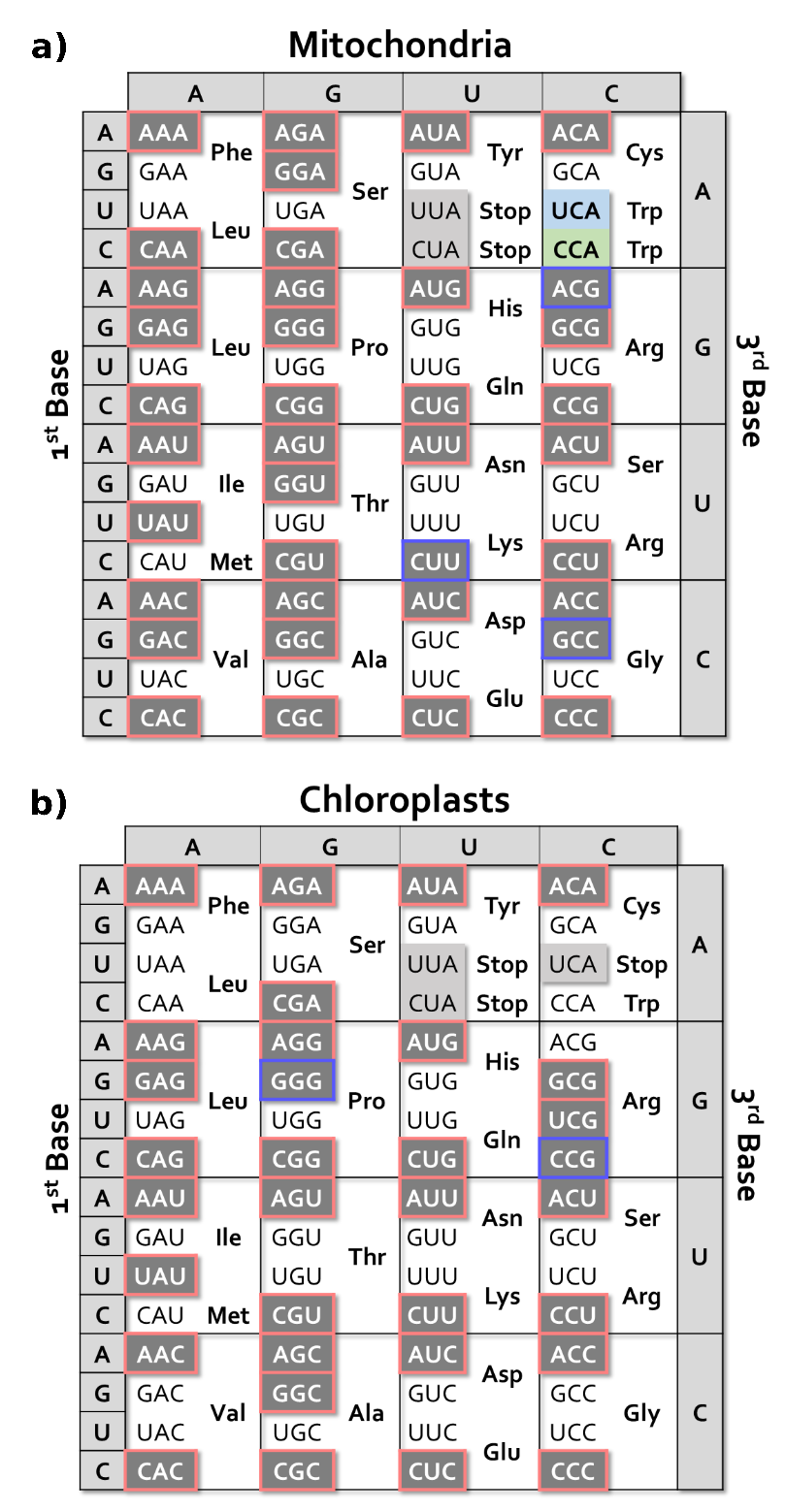


**Supplementary Figure 1.** Anticodon charts showing in dark gray background the missing isoacceptors in mitochondria (**a**) and in chloroplast (**b**) organelles. Red box perimeter indicates anticodons absent in 95-100% of the reported cases; purple box perimeter indicates anticodons absent in 85-95% of the cases. Sky-blue background corresponds to a Stop codon in plants and to a Trp anticodon in animals. Green background indicates a specific anticodon that recognizes a Trp codon in plants and is absent in animals.
